# FRAILTY ASSESSMENT FROM PAPER TO ONLINE: THE E-FI-CGA WEB APP

**DOI:** 10.1093/geroni/igad104.2248

**Published:** 2023-12-21

**Authors:** Xiaowei Song, Kiarash Kianpoor, Katayoun Sepehri, Stanley Kwok, Margit Glashutter, Robert McDermid, Grace Park

**Affiliations:** Fraser Health, Surrey, British Columbia, Canada; Fraser Health, Surrey, British Columbia, Canada; Fraser Health, Surrey, British Columbia, Canada; Fraser Health, Surrey, British Columbia, Canada; Fraser Health, Surrey, British Columbia, Canada; Fraser Health, Surrey, British Columbia, Canada; Fraser Health, Surrey, British Columbia, Canada

## Abstract

**Background:**

Early frailty assessment is crucial for improving care for older adults through enhancing frailty-informed care planning and execution. To increase the access and use of the established electronic Frailty Index based on the Comprehensive Geriatric Assessment tool, we developed an online software application: the web-based eFI-CGA.

**Methods:**

End-user requirements primarily adapted the standalone version of the eFI-CGA and covered assessment competency, interface familiarity, accessibility and convenient use by front-line health professionals. Web app-bounded features included user-account authentication and authorization, user-space management, record-search and retrieval, and functions for data management, analysis and display. The web app development used the Microsoft Azure web server with the ASP.NET framework, combined Webforms and Model-View-Controller architecture, and C# programming language with standard client-side libraries incorporating scripting and mark-up languages.

**Results:**

The web-based eFI-CGA was technically released and accessible online (efi-cga). Tests on the web pages for all the web pages (e.g., Home, Signup Login, Assessment, Search, and Analysis) using large sizes of systematically designed test data and mocked patient cohorts showed that the web software functions meet the requirements with 100% accuracy.

**Conclusion:**

The web-based eFI-CGA provides a valuable remote frailty assessment and information retrieval method for healthcare professionals, promoting effective multidisciplinary integrated care of older adults. Further work is needed to allow the safe use of the web app. We are also planning a patient-oriented frailty assessment tool for community-dwelling older adults.

